# Tau and TDP-43 synergy: a novel therapeutic target for sporadic late-onset Alzheimer’s disease

**DOI:** 10.1007/s11357-021-00407-0

**Published:** 2021-06-29

**Authors:** Caitlin S. Latimer, Nicole F. Liachko

**Affiliations:** 1 Department of Laboratory Medicine and Pathology, University of Washington, Seattle, WA 98195 USA; 2 Geriatrics Research Education and Clinical Center, Veterans Affairs Puget Sound Health Care System, GRECC, S182, 1660 South Columbian Way, Seattle, WA 98108 USA; 3 Division of Gerontology and Geriatric Medicine, Department of Medicine, University of Washington, Seattle, WA 98104 USA

**Keywords:** TDP-43, Tau, Amyloid-β (Aβ), Alzheimer’s disease, Limbic-predominant age-related TDP-43 encephalopathy (LATE), Frontotemporal lobar degeneration (FTLD)

## Abstract

Alzheimer’s disease (AD) is traditionally defined by the presence of two types of protein aggregates in the brain: amyloid plaques comprised of the protein amyloid-β (Aβ) and neurofibrillary tangles containing the protein tau. However, a large proportion (up to 57%) of AD patients also have TDP-43 aggregates present as an additional comorbid pathology. The presence of TDP-43 aggregates in AD correlates with hippocampal sclerosis, worse brain atrophy, more severe cognitive impairment, and more rapid cognitive decline. In patients with mixed Aβ, tau, and TDP-43 pathology, TDP-43 may interact with neurodegenerative processes in AD, worsening outcomes. While considerable progress has been made to characterize TDP-43 pathology in AD and late-onset dementia, there remains a critical need for mechanistic studies to understand underlying disease biology and develop therapeutic interventions. This perspectives article reviews the current understanding of these processes from autopsy cohort studies and model organism-based research, and proposes targeting neurotoxic synergies between tau and TDP-43 as a new therapeutic strategy for AD with comorbid TDP-43 pathology.

## Perspectives

### Pathophysiological spectrum in Alzheimer’s disease

Alzheimer’s disease (AD), a devastating, fatal neurodegenerative disease of aging, features the presence of extracellular amyloid-β (Aβ) plaques and intracellular tangles of hyperphosphorylated tau in the brain [1]. For decades, prevention or clearance of Aβ plaques have been the major therapeutic goals in the pursuit of a treatment for AD. This was a rational approach given that mutations in genes involved in amyloid processes cause some inherited (familial) cases of AD [2]. Unfortunately, most clinical trials have failed thus far to demonstrate a significant benefit to patients, raising the need to pursue additional strategies [3]. Familial mutations driving amyloidogenesis may represent a distinct form of AD in which amyloid pathology is both necessary and sufficient to trigger a cascade of events including pathological tau deposition and neurodegeneration. However, in aging brains that lack these genetic triggers for amyloid pathology, the Aβ plaques that accumulate may still be necessary but perhaps not sufficient to drive neurodegeneration. Accumulation of tau tangles strongly correlates with cognitive impairment and neurodegeneration in AD, and this recognition has stimulated a shift toward the development of tau-targeting therapies [4]. Although tau-targeting therapies hold great promise, to date only a few have made it to clinical trials [5].

**Fig. 1:**
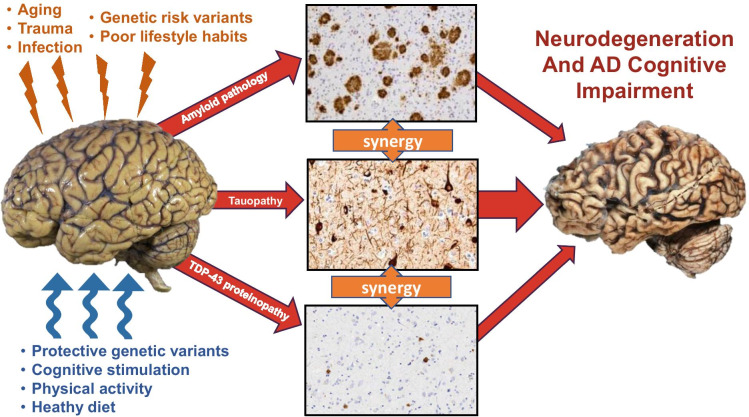
Pathways of neurodegeneration and cognitive impairment in AD. The human brain is subject to a variety of factors, both genetic and environmental, that either incite (orange) or mitigate (blue) the risk of developing the neuropathologic hallmarks of Alzheimer’s disease (amyloid plaques and neurofibrillary tangles). It is currently unknown whether these factors also influence the development of TDP-43 proteinopathy. While initiation of amyloid, tau, or TDP-43 pathology may occur through independent or overlapping events, the presence of multiple pathologies likely drives synergistic neurotoxicity that leads to cognitive decline and neurodegeneration. Observational data from human autopsy studies, as well as work from model systems, suggest that TDP-43 pathology enhances tau deposition and exacerbates the clinical severity of AD

While historically AD has been thought of as a single disease entity driven by dysfunction or accumulation of Aβ and tau, it may more accurately resemble a spectrum of diseases with variable but overlapping clinical and neuropathological features [6]. Amyloid plaques and tau tangles remain the defining pathologic constants of AD, but their respective contributions to neurodegeneration and clinical presentation may differ depending on other genetic, environmental, and pathological factors in play (Fig. 1). Rare mutations in amyloid processing genes cause AD, implicating Aβ in the disease pathophysiology. However, these mutations usually result in much earlier onset of disease distinct from the more common sporadic late-onset AD (LOAD). In LOAD, genome-wide association studies (GWAS) and genome sequencing studies have identified numerous alleles that modify AD risk [7], the strongest modifier being apolipoprotein E [8]. A variety of lifestyle and environmental factors are also believed to influence the risk of LOAD, including level of education, diet, and physical activity [9–12]. Furthermore, comorbid pathologies, such as vascular brain injury, Lewy body disease, or TDP-43-positive inclusions, are a common observation in the brains of older individuals.

One outstanding question is whether these pathologies have a contributory effect on AD neuropathology or the clinical expression of the disease [13–15]. In particular, TDP-43 has become increasingly recognized as a frequent co-pathology in the brains of individuals with the clinical and neuropathologic features of AD.

### TDP-43 clinicopathological correlates in Alzheimer’s disease

TDP-43, an essential protein, has a variety of critical cellular functions that include regulating pre-mRNA splicing, mRNA transport, and forming and stabilizing stress granules [16]. TDP-43 was first linked to neurodegenerative disease when it was identified as the major pathologic protein comprising ubiquitin-positive inclusions in the majority of amyotrophic lateral sclerosis (ALS) and approximately half of frontotemporal lobar degeneration (FTLD) cases [17, 18]. Mutations in the gene coding for TDP-43, *TARDBP*, cause ~ 4% of familial inherited ALS, directly linking TDP-43 dysfunction with disease [19, 20]. Pathological TDP-43 has since been described in association with numerous other neurodegenerative diseases, most notably Alzheimer’s disease, where it is present in up to 57% of cases in some autopsy cohorts [21–23]. In these cohorts, TDP-43 pathology is found in the limbic structures of older individuals with AD neuropathologic change (ADNC) [24–27]. TDP-43 often associates with hippocampal sclerosis [28, 29], and while it can occur in the absence of AD pathology, particularly in the oldest old (90 +), this is much less common and of uncertain clinical significance [30, 31]. Individuals with both ADNC and TDP-43 pathology typically have more severe cognitive impairment, faster rates of decline, and more rapid hippocampal atrophy over time [24, 32]. At the opposite end of the spectrum, a rarer number of so-called “resilient” individuals develop some degree of amyloid and tau pathology but are able to maintain their cognitive function [33–35]. Recent work suggests that one such factor determining resilience versus susceptibility to AD may be the presence of pathological TDP-43 [24, 33, 36, 37].

In a small but well-characterized group of resilient individuals with ADNC from the Adult Changes in Thought (ACT) autopsy cohort, the absence of TDP-43 distinguished those without cognitive impairment (resilient) from those with cognitive decline (AD dementia). When compared to individuals with dementia and matched levels of ADNC, resilient individuals did not have any differences in Lewy bodies or vascular brain injury (including arteriolosclerosis, atherosclerosis, macro- and micro-infarcts, and cerebral amyloid angiopathy). The key difference between the resilient and AD dementia cohorts was the presence of TDP-43 pathology, indicating aberrant or aggregated TDP-43 may be a significant contributing factor to developing dementia [33]. Other community-based cohorts have demonstrated similar associations between the presence of TDP-43 and the likelihood of dementia. In fact, staging criteria for TDP-43 pathology in AD has been developed, with higher stages correlating with worse cognitive impairment [24, 38, 39]. These associations are intriguing but raise new questions. It is currently unknown why some individuals with AD develop TDP-43 pathology while others do not, or why clinical manifestations of AD are more severe in individuals with TDP-43 pathology. Autopsy studies suggest the presence of TDP-43 pathology may lower the threshold for developing the clinical symptoms of AD [26]. However, additional research is needed to understand the mechanisms underlying TDP-43 deposition and AD phenotypes.

### Evidence for synergism among TDP-43, Aβ, and tau

In addition to studying the correlations between TDP-43 and cognitive decline, cohort studies have also evaluated the relationship between TDP-43 and the neuropathologic hallmarks of AD, amyloid and tau pathology. Data from the Religious Orders Study and the Memory and Aging Project (ROS/MAP) inform models that implicate amyloid pathology, particularly neuritic plaques, in TDP-43 pathology [40]. The relationship between Aβ and TDP-43 is not well understood. Given the observation that TDP-43 co-pathology is much more common in the presence of ADNC than in pure tauopathies such as FTLD-tau, progressive supranuclear palsy, or corticobasal syndrome, there may be an interaction between amyloid and TDP-43. There is some limited evidence supporting this hypothesis. In vitro, TDP-43 does not regulate expression or processing of amyloid precursor protein but accelerates Aβ aggregation, and in mouse models, it modulates Aβ fibrillization and worsens cognitive outcomes [41–43]. However, in an autopsy cohort of AD patients who underwent antemortem amyloid sensitive PET imaging, there were no correlates between TDP-43 pathology and global amyloid PET signal [44]. It is possible there is a reciprocal effect where Aβ also influences the initiation or development of TDP-43 pathology, although this remains to be determined.

In other AD cohorts, there is evidence for a relationship between TDP-43 and tau pathology, which appear to develop in a similar pattern throughout the brain, beginning in limbic structures and later advancing to neocortex [21]. Josephs et al. at the Mayo Clinic describe higher Braak stage of tau pathology in subjects with comorbid TDP-43 pathology [23], and in the ACT study it was shown that for individuals matched by Braak stage, those with TDP-43 co-pathology had a higher density of tau pathology in frontal cortex [33]. Although different assessments of tau pathology were used in these two studies, this complementary data from two different cohorts suggest that the presence of TDP-43 pathology is associated with increased tau burden. Additional studies have found that a subset of tau and TDP-43 positive inclusions are co-present or co-localized in the same neurons in AD brains [45, 46]. In addition, AD brain–derived phosphorylated tau and TDP-43 can co-immunoprecipitate indicating a strong physical interaction [45, 47, 48]. Given the previously described associations between tau pathology and cognitive dysfunction, a pathological relationship between tau and TDP-43 may underlie the increase in severity of cognitive symptoms when comorbid TDP-43 is identified in AD. Despite these associations, however, human cohort studies cannot be used to determine what the mechanisms of this relationship are, including whether TDP-43 enhances tau pathology, or vice versa. In addition, it remains to be determined whether development of TDP-43 pathology represents an early event in AD pathogenesis or occurs later as disease progresses. For this, it is necessary to develop and study models that can recapitulate both tau and TDP-43 proteinopathies simultaneously.

The models used to study Alzheimer’s disease focus on amyloid and tau pathology, either alone or in combination [49]. TDP-43 proteinopathy has predominantly been studied as a mono-proteinopathy modeling ALS and FTLD [50]. Only recently has TDP-43 proteinopathy been considered an important contributing variable in AD, and to date there are limited studies exploring the relationship between tau and TDP-43 in vitro or in vivo. Data generated by our lab using a *C. elegans* co-expression model of tau and TDP-43 found that tau and TDP-43 synergize leading to increased pathological protein accumulation, neuronal dysfunction, and neurodegeneration [33]. In cell and mouse models, TDP-43 regulates mRNA splicing of tau exon 10, shifting the ratio of tau microtubule-binding repeats from the normal balanced ratio of 3R/4R-tau to a higher proportion of 4R-tau [51]. Recent work found that tau oligomers promote accumulation of cytoplasmic TDP-43 in HEK293 cells, and brain-derived TDP-43 oligomers can cross-seed tau aggregates in vitro [52]. Finally, in transgenic rats expressing human familial ALS-mutant TDP-43(M337V), hippocampal injections of AAV9 virus carrying phosphorylation-mimic human tau(T175D) led to significantly increased burden of tau pathology than control animals with either TDP-43(M337V) or tau(T175D) alone [53]. Although additional studies are necessary, the current data supports a biological relationship between tau and TDP-43 that drives neurodegeneration in sporadic LOAD. Indeed, if a biomarker of TDP-43 pathology could be developed, this may prove to be a crucial addition to the A,T(N) research framework that currently exists for characterizing risk of dementia [54].

### Potential mechanisms of tau and TDP-43 synergy

**Fig. 2:**
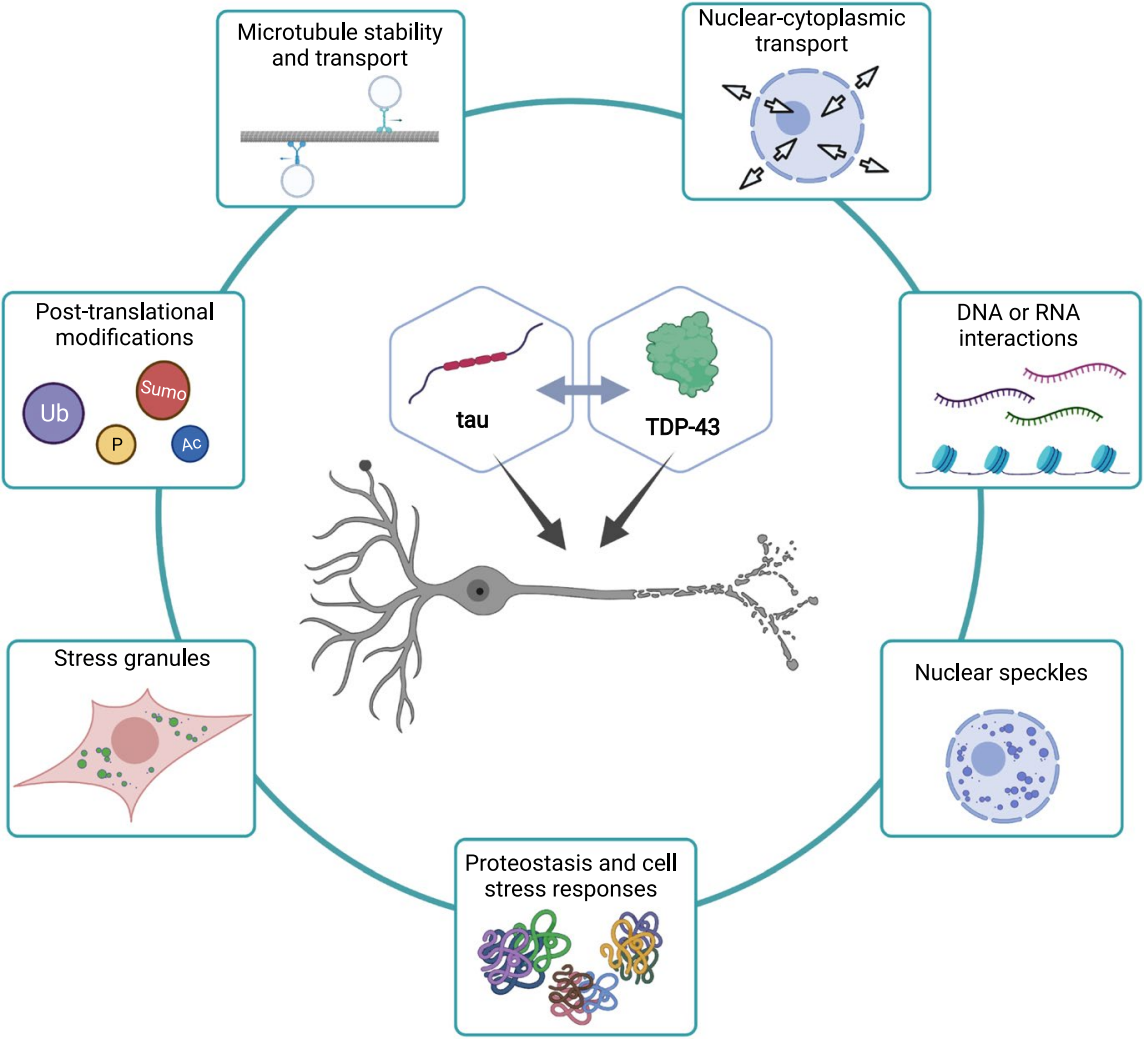
Potential mechanisms for tau and TDP-43 synergy in AD**.** There are numerous pathways that potentially underlie tau and TDP-43 synergistic neurotoxicity. These include interactions within the nucleus during regulated transport between the nucleus and cytoplasm, or associations with DNA, RNA, or nuclear speckles. In the cytoplasm, alterations in microtubule dynamics, stability, or transport along microtubules, derangement of cellular signaling including post-translational modifications such as phosphorylation, ubiqitination, acetylation, or SUMOylation, or formation of stress granules could lead to phenotypic enhancement of tau and TDP-43. Finally, misfolded tau or TDP-43 protein can impair normal cellular proteostasis mechanisms, which may have a broader impact on other aggregation prone proteins

While the observations from both human cohorts and limited studies using model systems suggest an interaction between tau and TDP-43, the mechanisms that underlie that interaction are unknown. However, there is a wealth of information known about the physiological roles of both tau and TDP-43, as well as their respective roles in other neurodegenerative disease, that serve as a guide and potential place to start the investigation. As a component of tubulin assembly, tau binds and stabilizes microtubules. Tau localizes throughout the cytoplasm, and is enriched in axons, where it assists with cargo trafficking along the microtubule network [55]. Phosphorylation of tau decreases its microtubule-binding affinity, resulting in destabilization of microtubules. Although predominantly found in the cytoplasm, tau is also present in the nucleus where it can stabilize heterochromatin [56]. Recently, tau aggregates have been found to include components of nuclear speckles, which are protein and RNA-containing structures within the nucleus [57]. TDP-43 has demonstrated roles in DNA binding, transcription regulation, pre-mRNA splicing of the majority of transcripts, alternative splice site selection, mRNA stability and transport, and microRNA biogenesis [58]. TDP-43 is typically nuclear, but it can be transported to the cytoplasm. There are numerous possibilities for biological or disease-state interactions between tau and TDP-43 throughout the cell (Fig. 2). In the nucleus, this could extend to DNA or RNA associating functions of tau and TDP-43 including mRNA splicing regulation, nuclear speckle activities, or nuclear-cytoplasmic transport [57, 59–64]. In the cytoplasm, TDP-43 may impact tau-associated microtubule stability and transport by binding dynactin [65]. Furthermore, cellular reactions to pathological tau or TDP-43 alone, including formation of stress granules, post-translational modification based signaling such as kinase activation, or changes in proteostasis pathway dynamics, may promote dysfunction and synergistic neurotoxicity from the other protein.

## Conclusions and future studies

A majority of patients with sporadic late-onset AD exhibit comorbid TDP-43 pathology, which strongly correlates with cognitive outcomes. The presence of pathologic TDP-43 may enhance tau pathology and concomitant neurotoxicity, either independent of or in concert with amyloid pathology. If true, this may help explain the limited efficacy of amyloid focused therapeutics and argue for the need to develop a multitargeted approach to preventing tau progression and subsequent neurodegeneration. However, while there are clearly intriguing associations observed between tau and TDP-43 in human autopsy and model organism studies, additional work is needed to broaden understanding of these processes. Some suggested areas for future study are described below.

### Human cohort studies

Additional community-based cohort studies that include cognitive assessments in life paired with complete neuropathological examination after death are need to supplement the published literature, which currently relies on relatively few cohorts. There are several detailed examinations of TDP-43 pathology described in the literature [38, 39, 45, 66]. However, additional descriptions of the regional distribution, cell type vulnerability, cell compartment localization, and biochemistry of TDP-43 pathology are necessary to better characterize TDP-43 in the context of ADNC, and if possible, distinguish it from other TDP-43 proteinopathies. There is also a need to develop biomarkers for TDP-43 proteinopathy in order to identify these individuals during life, rather than requiring an autopsy. Furthermore, transcriptomic or proteomic studies from these human cohorts will be imperative to identify underlying mechanisms and pathophysiological pathways. Finally, with the addition of more cohorts with pathologically confirmed AD and TDP-43 pathology, GWAS can be performed to identify underlying genetic risk factors that may predispose an individual to developing combined tau, Aβ, and TDP-43 pathology. As we better characterize this disease in humans clinically, pathologically and genetically, progress can be made to generate model system that more accurately represent the disease and used to probe potential pathways and molecular mechanisms.

### Model systems

Further development of model organisms that express multiple pathologic proteins is necessary, including those that combine amyloid, tau, and TDP-43 pathologies. Early work in model systems suggests that the presence of TDP-43 exacerbates tau neurotoxicity, worsening disease phenotypes. Therefore, TDP-43 may be a highly relevant therapeutic target. However, these models are only beginning to emerge. It will be necessary to integrate data from human studies into the development of more refined model systems, allowing dissection of interactions between tau and TDP-43. These models should include simple cell culture or invertebrate systems allowing large-scale screening and rapid progress, as well as vertebrate models to investigate disease relevant phenotypes in a mammalian brain. Studies should incorporate consideration of aging as a factor influencing disease. Additionally, conditional expression models will be necessary to study the temporal relationships between tau and TDP-43 and better understand whether perturbation of one is upstream and capable of inducing pathology of the other. Finally, models co-expressing all three proteins (Aβ, TDP-43, and tau) are also necessary to better understand how TDP-43 fits into the A,T(N) framework. As understanding of the pathological role of TDP-43 in AD progresses, essential future work will be to develop therapeutic strategies that prevent or remove disease-promoting TDP-43 and restore healthy brain function.

Overall, determining the contributory roles TDP-43 plays in the progression of AD will require combining autopsy studies of the human pathology with large-scale human genetics and model organism-based experiments. The rewards are great, as determining the nature of TDP-43 contributions to AD may open up a novel path in the pursuit of AD therapeutics.
